# Childhood Cancer Incidence and Survival in South Australia and the Northern Territory, 1990–2017, with Emphasis on Indigenous Peoples

**DOI:** 10.3390/cancers16112057

**Published:** 2024-05-29

**Authors:** Suzanne Mashtoub, Shahid Ullah, Anne Collinson, Gurmeet R. Singh, Justine Clark (Adnyamathanha), Shalem Leemaqz, Ora Paltiel, David M. Roder, Benjamin Saxon, Ross McKinnon, Stephen J. Pandol, Claire T. Roberts, Savio George Barreto

**Affiliations:** 1 Department of Surgery, Flinders Medical Centre, Bedford Park, Adelaide, SA 5042, Australia; suzanne.mashtoub@flinders.edu.au; 2 College of Medicine and Public Health, Flinders University, Bedford Park, SA 5042, Australia; shahid.ullah@flinders.edu.au (S.U.); shalem.leemaqz@flinders.edu.au (S.L.); ross.mckinnon@flinders.edu.au (R.M.); 3 Faculty of Health and Medical Sciences, University of Adelaide, Adelaide, SA 5000, Australia; 4 Royal Darwin Hospital, Darwin, NT 0810, Australia; collinson.anne@gmail.com; 5 Menzies School of Health Research, Charles Darwin University, Darwin, NT 0800, Australia; gurmeet.singh@menzies.edu.au; 6 Indigenous Genomics, Telethon Kids Institute, Adelaide, SA 5000, Australia; justine.clark@telethonkids.org.au; 7 John Curtin School of Medical Research, Australian National University, Canberra, ACT 2601, Australia; 8 Braun School of Public Health, Hebrew University of Jerusalem, Jerusalem 9112102, Israel; orap@hadassah.org.il; 9 Department of Hematology, Faculty of Medicine, Hebrew University of Jerusalem, Jerusalem 9112102, Israel; 10 Cancer Epidemiology and Population Health, UniSA Allied Health and Human Performance, Adelaide, SA 5001, Australia; david.roder@unisa.edu.au; 11 Department of Haematology/Oncology, Women’s and Children’s Hospital, Adelaide, SA 5000, Australia; ben.saxon@sa.gov.au; 12 Paediatric Education, University of Adelaide, Adelaide, SA 5005, Australia; 13 Flinders Health and Medical Research Institute, Adelaide, SA 5042, Australia; 14 Division of Digestive and Liver Diseases, Cedars-Sinai Medical Center, Los Angeles, CA 90048, USA; stephen.pandol@cshs.org

**Keywords:** outcomes, morbidity, mortality, children, Indigenous

## Abstract

**Simple Summary:**

Reports of a significant increase in young-onset cancers in South Australia (SA) with a higher-than-average incidence in the Northern Territory (NT) in the last 3 decades. Cancer incidence significantly decreased over time amongst non-Indigenous children but remained unchanged amongst Indigenous children (NT). Overall survival improved in SA, but unchanged in the NT. In the NT, overall survival of Indigenous children was significantly lower than non-Indigenous children, but survival rates in the former have improved. Ongoing targeted public health response is needed to reduce disparities in cancer health care outcomes for Indigenous Australians as outlined within the Australian and the Aboriginal and Torres Strait Islander Cancer Plans.

**Abstract:**

Background & Aims: Reports of a rise in childhood cancer incidence in Australia and globally prompted the investigation of cancer incidence and survival in South Australia (SA) and the Northern Territory (NT) over a 28-year period, with emphasis on Indigenous peoples. Methods: This cross-sectional analysis of two prospective longitudinal databases, the SA and NT Cancer Registries (1990–2017), included all reported cases of childhood cancers. Poisson regression provided estimates of incidence rate ratios and survival was modelled using Cox proportional hazard models for children aged <5 and ≥5 years. Results: A total of 895 patients across SA (*N* = 753) and the NT (*N* = 142) were ascertained. Overall and in the NT, childhood cancer incidence was higher in males compared with females (IRR 1.19 [1.04–1.35] and 1.43 [1.02–2.01], respectively). Lymphocytic leukemia was the most reported cancer type across all locations. With reference to the 1990–1999 era (181.67/100,000), cancer incidence remained unchanged across subsequent eras in the combined cohort (SA and NT) (2000–2009: 190.55/100,000; 1.06 [0.91–1.25]; 2010–2017: 210.00/100,000; 1.15 [0.98–1.35]); similar outcomes were reflected in SA and NT cohorts. Cancer incidence amongst non-Indigenous children significantly decreased from the 1990–1999 era (278.32/100,000) to the 2000–2009 era (162.92/100,000; 0.58 [0.35–0.97]). Amongst 39 Indigenous children in the NT, incidence rates remained unchanged across eras (*p* > 0.05). With reference to the 1990–1999 era, overall survival improved in subsequent eras in SA (2000–2009: HR 0.53 [0.38–0.73]; 2010–2017: 0.44 [0.28–0.68]); however, remained unchanged in the NT (2000–2009: 0.78 [0.40–1.51]; 2010–2017: 0.50 [0.24–1.05]). In the NT, overall survival of Indigenous patients was significantly lower compared with the non-Indigenous cohort (3.42 [1.92–6.10]). While the survival of Indigenous children with cancer significantly improved in the last two eras (*p* < 0.05), compared to the 1990–1999 era, no change was noted amongst non-Indigenous children in the NT (*p* > 0.05). Conclusions: The incidence of childhood cancers has remained unchanged over 28-years in SA and the NT. Encouragingly, improved survival rates over time were observed in SA and amongst Indigenous children of the NT. Nevertheless, survival rates in Indigenous children remain lower than non-Indigenous children.

## 1. Introduction

In recent decades, there has been a concerning increase in global reports of cancers affecting children and young adults [1,2,3]. Recent interrogation of Australian data, derived from the South Australian (SA) and Northern Territory (NT) Cancer Registries (SACR and NTCR), revealed an increase in young-onset adult gastrointestinal adenocarcinomas in SA [4] and a higher-than-average incidence rate in the NT since 1990 [5]. Moreover, the overall incidence rate of childhood cancer in Australia increased by 34% between 1983 and 2015, rising by 1.2% yearly between 2005 and 2015, with an expected 7% rise by 2035 [6].

The underlying causes for early-onset carcinogenesis have largely remained speculative, implicating exposure to early antibiotics and alteration of gut microbiome, lifestyle factors including cigarette smoking, alcohol, physical inactivity and rising incidence of obesity, variations in mismatch repair genes and microsatellite instability [7,8,9,10,11]. Social determinants of health also influence cancer risk, resulting in significant health inequities between populations. This is particularly evident among worldwide Indigenous populations, attributed to the ongoing impacts of colonization and marginalization [12,13,14]. Cancer disproportionately impacts Aboriginal and Torres Strait Islander peoples, herein respectfully referred to as Indigenous Australians, who are 40% more likely to die of cancer in comparison to non-Indigenous Australians [15]. Indigenous Australians represent 3.3% of the total Australian population, but 26.3% of the NT population [16]. Over 79% of the Australian population resides in its Eastern states and territories, whereas SA and NT represent some of the lowest populated states and territories with low population densities, resulting in unique challenges to cancer care delivery [16].

It was hypothesized that the incidence of childhood cancers is increasing in SA and the NT. We aimed to determine the age and incidence of childhood cancers (leukemias and embryonal tumors) and characterize the trends in overall survival over a 28-year period in SA and the NT. Additionally, we aimed to gain insight into the trends and overall survival amongst Indigenous Australian children afflicted with cancer in the NT. The findings of the current study will support the ongoing monitoring of cancer outcomes for children in SA and NT, which can be leveraged to influence improved policy and clinical practice, particularly for NT Indigenous Australian children affected by cancer.

## 2. Methods

A retrospective analysis of longitudinal data, collected prospectively from 1 January 1990 to 31 December 2017, was undertaken. Data was acquired from both SACR and NTCR, focusing on all cases of lymphocytic leukemia, nephroblastoma, neuroblastoma, Ewing sarcoma, osteosarcoma, retinoblastoma, hepatoblastoma, rhabdomyosarcoma and medulloblastoma.

All cases of invasive cancer are notifiable via the Health Care (Reporting of Cancer) Regulations under the SA *Health Care Act 2008* [17]; SACR has been collecting data on cancer incidence and mortality since 1977 and is managed by Wellbeing SA’s Epidemiology Branch (under the auspices of SA Health). Similarly, the NTCR, established in 1981 by the NT Department of Health and Families, captures all NT cancer diagnoses and cancer-related deaths in accordance with the NT Cancer (Registration) Regulations under the NT *Cancer (Registration) Act 2011* [18].

Ethics approval was obtained from the SA Department for Health and Wellbeing Human Research Ethics Committee (HREC; Reference number 2021/HRE00174) and the HREC and Aboriginal Ethics Sub-Committee of the NT Department of Health and Menzies School of Health Research (HREC Reference Number 2021-4027). A waiver of consent was provided by both HRECs, considering only de-identified data were obtained.

### 2.1. Selection of Cases

#### 2.1.1. Inclusion Criteria

Cases of SA and NT residents aged 19 years of age and younger with a pathologically confirmed diagnosis of lymphocytic leukemia, nephroblastoma, neuroblastoma, Ewing sarcoma, osteosarcoma, retinoblastoma, hepatoblastoma, rhabdomyosarcoma and medulloblastoma were included. Histology codes included International Classification of Diseases (ICD) C64.9, C91.0, C91.01, C91.02, C92.0, C92.01, C92.02, C71.6, C74.90, C22.2, 9510/2, 9511/3, 9512/3, 9513/3, 9514/1, 9186/3, 9260/3, 8902/3. The study period was categorized into three time periods (eras 1990–1999, 2000–2009, 2010–2017) to reflect incidence and survival of cancers over time.

#### 2.1.2. Statistical Analysis

All statistical analyses were conducted using R version 4.2.3 (R Core Team, 2023) and Stata version 16.1 (StataCorp, 2019). Patient characteristics were expressed as median and interquartile range (IQR) for skewed data. The Mann-Whitney U test was used to explore the significance of differences in patients’ age between two groups of patients. Proportions were presented as percentages of the respective denominator and were compared between groups using a standard Chi-square test for association with continuity correction, where appropriate.

The incidence rates (IR) were calculated by taking the total number of cases divided by the population at risk. Age-sex specific population data were extracted from 1990–2017 and divided by the respective average population. Specifically, the incidence rate among the <5-year age group in SA was determined by dividing the total number of cases in that age group in SA by the average population spanning 28 years, from 1990 to 2017, for that age group in SA. This same methodology was employed for gender and cancer subtype analyses. However, for era-specific incidence rates, the total number of cases within a particular era was divided by the average population of that era. The 2011 population census data were used to calculate the incidence rates between Indigenous and non-Indigenous populations in the NT. The rates were presented per 100,000 persons over three time periods for age groups <5 years and ≥5 years, for each sex and cancer primary subtypes. A Poisson regression model was applied to examine the incidence rates between groups of the above characteristics. Estimates were calculated using the likelihood ratio method and expressed as incidence rate ratios (IRRs) from the Poisson regression model. The IRRs were considered statistically significant if their 95% CI did not include unity. The more the IRR deviated from 1, the stronger the association between the exposure variable and outcome. Residual deviance was used to evaluate the model goodness of fit.

Survival was measured from the date of cancer diagnosis to the date of death or individuals were censored at date of loss to follow-up or census date; the census date was assigned on 31st December 2017. Five-year survival rates were calculated for each cancer subtype across state levels and Indigenous status. Survival was evaluated by examining standard Kaplan-Meier survival curves and patient cohorts were compared by the log-rank test. Cox proportional hazards models were applied to examine survival outcomes by modelling the age-specific incidence rate of death given other factors. Age, sex, primary subtypes, and cohort era were used to explore the risk of death. The estimates were calculated using the likelihood ratio method and were expressed as hazard ratios (HRs); the lower the HR, the longer the survival. Proportional hazard assumption was tested by log-log plot of survival and Schoenfeld Residuals. A Harrell’s C-statistics was used to explore the predictive discrimination ability of the model. The two-sided test was performed for all analyses, 95% confidence intervals (CI) were reported, and the level of significance was set at α = 0.05.

## 3. Results

### 3.1. Patient Demographics and Reported Cancers

A total of 895 patients across SA (*N* = 753) and NT (*N* = 142) were diagnosed with lymphocytic leukemia, nephroblastoma, neuroblastoma, Ewing sarcoma, osteosarcoma, retinoblastoma, hepatoblastoma, rhabdomyosarcoma or medulloblastoma between 1990 and 2017 (Table 1). The median ages for the combined SA and NT cohort, SA only and NT only cohorts were five (IQR 2–13), four (2–12) and seven years (2–16), respectively.

The overall IR was 193.04 (95%CI 180.60–206.12) per 100,000 residents across the combined cohort (SA and NT), 189.37 (176.09–203.39) in SA alone and the highest in the NT at 215.16 (181.23–253.60; Table 2). With reference to the 1990–1999 era (181.67/100,000), cancer incidence remained unchanged across subsequent eras in the combined cohort (SA and NT) (2000–2009: 190.55/100,000; 1.06 [0.91–1.25]; 2010–2017: 210.00/100,000; 1.15 [0.98–1.35]); similar outcomes were reflected in SA and NT cohorts (Table 2). Importantly, a significantly higher incidence was noted in the <5 years group (SA and NT 379.02/100,000; SA 391.99/100,000; NT 309.97/100,000) compared with individuals aged ≥5 years (SA and NT 132.23/100,000, IRR 0.35, 95%CI 0.31–0.40, *p* < 0.001; SA 124.73/100,000, IRR 0.32, 0.28–0.37, *p* < 0.001; NT 179.42/100,000, IRR 0.58, 0.41–0.81, *p* < 0.01; Table 2). The incidence was higher in males (208.92/100,000) compared with females (176.31/100,000) in the combined SA and NT cohort (IRR 1.19, 1.04–1.35, *p* < 0.01) and NT cohort (male 175.91/100,000; female 175.91/100,000; IRR 1.43, 1.02–2.01, *p* < 0.05). In SA, there was no significant difference in IRR (1.14, 0.99–1.32, *p* = 0.07) based on sex (male 201.74/100,000; female 176.38/100,000; Table 2, Supplementary Figure S1).

Lymphocytic leukemia was the most reported cancer type across all locations (SA and NT 119.71/100,000; SA 121.47/100,000; NT 109.10/100,000; Table 2). It was noted to be significantly more common in <5-year-olds versus ≥5 years in SA (IRR 0.40, 95%CI 0.33–0.48; *p* < 0.001) and the NT (0.50, 0.31–0.79, *p* < 0.01; Supplementary Figure S2). In SA, overall survival of children diagnosed with lymphocytic leukemia significantly improved in the 2000–2009 and 2010–2017 eras, compared with 1990–1999 (*p* < 0.001), whereas in the NT, survival significantly improved (*p* < 0.01) only in the most recent era (2010–2017) compared with 1990–1999 (Supplementary Figure S3). Medulloblastoma was the least commonly reported cancer subtype (SA and NT 1.94/100,000; SA 1.76/100,000; NT 3.03/100,000). Each cancer subtype was largely consistent in incidence across the two locations; however, the incidence of rhabdomyosarcoma was greater in the NT (36.37/100,000) compared with combined SA and NT cohort (8.2/100,000) and SA alone (3.52/100,000, *p* < 0.001; Table 2).

### 3.2. Non-Indigenous and Indigenous Patient Demographics and Reported Cancers in NT

In the NT, a total of 139 children, comprising 100 non-Indigenous and 39 Indigenous children, were diagnosed with cancer subtypes (Table 1). The overall IR for non-Indigenous children was 226.28 (95%CI 184.11–275.22) compared with 158.94 (113.02–217.28) for Indigenous children. The <5-year-olds had significantly greater incidence of childhood cancer compared with ≥5 years group in both non-Indigenous (IRR 0.66, 0.44–0.99, *p* = 0.046) and Indigenous (0.43, 0.22–0.81, *p* < 0.01) cohorts (Table 3). The incidence rates did not significantly vary by sex in non-Indigenous (*p* = 0.06) and Indigenous cohorts (*p* > 0.05). In the non-Indigenous cohort, the IRR significantly decreased from the 1990–1999 era (IR 278.32/100,000, 199.73–377.58) to the 2000–2009 era (162.92/100,000, 104.38–242.41; IRR 0.58, 0.35–0.97, *p* < 0.05), with no significant change in 2010–2017 (237.59/100,000, 165.49–330.44, IRR 0.85, *p* > 0.05; Table 3). Amongst Indigenous children, incidence rates remained statistically unchanged across eras (1990–1999: 110.04/100,000, 50.32–208.89; 2000–2009: 195.62/100,000, 111.82–317.68; 2010–2017, 171.17/100,000, 93.58–287.19, *p* > 0.05; Table 3).

Lymphocytic leukemia remained the most reported cancer subtype in both non-Indigenous (101.83/100,000, 95%CI 74.27–136.25) and Indigenous (105.96/100,000, 69.22–155.26) children in the NT, with medulloblastoma representing the least reported cancer subtype (non-Indigenous 2.26/100,000, 0–12.61; Indigenous 4.08/100,000, 0–22.71; Table 3). The incidence of rhabdomyosarcoma was 52.04/100,000 (32.99–78.09) in the non-Indigenous cohort, with no cases reported in Indigenous peoples during the study period (Table 3).

### 3.3. Survival by Time Trends and Subtype

The 5-year survival rates in SA (77%, 95%CI 74–80) and the NT (62%, 95%CI 51–71) were similar. By cancer subtypes in SA, lymphocytic leukemias, nephroblastoma, hepatoblastoma and rhabdomyosarcoma revealed greater 5-year survivals compared with other cancer types, with similar survivals observed in the NT (Table 4; Tables S1–S3).

Overall survival was significantly lower in the ≥5-year group versus the <5-year reference group in SA (HR 2.00, 1.47–2.72, *p* < 0.001; Table 5), whereas in the NT, survival did not differ significantly between age groups (HR 1.57, 0.84–2.94, *p* > 0.05). There was no difference in overall survival between males and females in both SA (HR 0.98, 0.73–1.32, *p* > 0.05) and the NT (HR 0.93, 0.52–1.66, *p* > 0.05). With reference to the 1990–1999 era, overall survival improved in subsequent eras in SA (2000–2009: HR 0.53, 0.38–0.73, *p* < 0.001; 2010–2017: HR 0.44, 0.28–0.68, *p* < 0.001); however, remained unchanged in the NT (2000–2009: HR 0.78, 0.40–1.51; 2010–2017: HR 0.50, 0.24–1.05, *p* > 0.05; Table 5).

### 3.4. Survival by Time Trends and Subtype in Non-Indigenous Compared with Indigenous Children in the NT

Among non-Indigenous children in the NT, the 5-year survival was 79% (95%CI 69–85), while for the Indigenous cohort, it was notably lower at 38% (95%CI 22–53; Table 6). Survivals exceeded 70% for all cancer subtypes in the non-Indigenous group, except for Ewing Sarcoma and osteosarcoma. In contrast, all cancer subtypes in the Indigenous Australian cohort had survivals below 70% (Table 6). In the NT, overall survival of Indigenous Australian children was lower compared with the non-Indigenous cohort (HR 3.42, 1.92–6.10, *p* < 0.001). No difference in survival was noted between <5 and ≥5-year-old non-Indigenous and Indigenous Australian children (*p* > 0.05). While the survival of Indigenous Australian children with childhood cancers has significantly improved in the last two eras (2000–2009: HR 0.35, 0.13–0.93, *p* = 0.04; 2010–2007: HR 0.29, 01.0–0.83, *p* = 0.02; Table 7) compared to the 1990–1999 era, no significant change was noted amongst non-Indigenous children (*p* > 0.05). Overall, no difference in survival was observed between male and female children, irrespective of their Indigenous status (*p* > 0.05).

## 4. Discussion

This study investigated the incidence and survival of childhood cancers in SA and the NT between 1990 and 2017, with further insights into cancer trends amongst Indigenous and non-Indigenous children in the NT.

The incidence of childhood cancers in SA and the NT has remained largely unchanged over 28-years. Additionally, incidence rates were lower in children aged ≥5-years compared with their younger counterparts; this effect was less pronounced in the NT. According to data derived from the International Classification of Childhood Cancer 3rd Edition, the rates of cancer from 2011 to 2015 in Australian children aged zero to 14 were slightly higher (16.3/100,000) than for the period between 2006 and 2010 (15.1/100,000) [19]. Furthermore, whilst rates increased for all age groups, the largest increase was for children aged five to nine (2006–2010: 10.3/100,000; 2011–2015: 12.3/100,000) [19]. On a global scale, an increase in the world age-standardized rate for all cancer incidence in children aged zero to 14 years from the 1980s to the period between 2001 and 2010 was noted; an increase observed in all regions, except sub-Saharan Africa [20].

It was postulated that increased global rates of childhood cancer since the 1980s may be attributed to improved diagnostic strategies, the ongoing development of cancer registration and more effective ascertainment techniques [19,20]. However, it is the perinatal period [21] that may help uncover the factors underlying young-onset carcinogenesis. Barreto and Pandol postulated the PELICan hypothesis for young-onset carcinogenesis; insults in utero trigger epigenetic and hormonal modifications to aid fetal adaptation and survival, with subsequent exposure to similar stressors in early childhood prompting further epigenetic modifications, resulting in premature activation of driver mutations [1,2]. The absence of a change in incidence of childhood cancers over time, especially in SA in which a significant increase in young-onset cancers was documented over the same period [4], is pertinent.

Consistent with global data [20], lymphocytic leukemia was the most reported childhood cancer across both SA and the NT, with lowest reports of medulloblastoma in both regions. The incidence of rhabdomyosarcoma was significantly greater in the NT compared with SA, with no significant variation in other cancer subtypes across locations. Male sex was associated with higher rates of childhood cancer incidence compared with females in the NT, with no significant sex differences in SA. Population-based studies from the United States have indicated a higher incidence rate of childhood cancer in males in general and by tumor type [22]. Differences in gene expression from autosomes or the X chromosome, rather than birthweight, may underlie sex differences in childhood tumor risk [22].

Overall survival from childhood cancers has significantly improved in SA, though not in the NT. A retrospective, population-based cohort study, extracting case information from the Australian Childhood Cancer Registry between 1983 to 2016 was undertaken [23]. Data indicated that Australian children diagnosed with cancer between 1983 and 1994 were almost twice as likely to die within five years compared with children diagnosed between 2007 and 2016, with corresponding relative survival rates of 73% and 86%, respectively [23]. These gains in survival translated to an estimated 1500 avoided deaths between 1995 and 2016, representing greater than one-third of all expected deaths within five years of diagnosis [23]. Akin to the current study outcomes, there were no differences in survival by sex for childhood cancers [23]. On a global scale, the five-year survival reports for all childhood cancers combined in Australia (86%: 2007–2016) was comparative with the United States (85%: 2011–2017) [24] and England (84%: 2011–2015) [25]; however, these evaluations must be interpreted with caution as they do not account for differences in the range of cancers analyzed between countries. Considering there has been minimal change in the distribution of stage at diagnosis for Australian children with cancer, except retinoblastoma and hepatoblastoma, improved survival over the past two decades reflects the implementation of more effective therapies derived from large clinical trials [26].

Indigenous Australians represent 3.3% of the total Australian population, but 26.3% of the NT population [16]. Indigenous Australians experience barriers in accessing cancer health care stemming from geographic remoteness of residence, low socio-economic status, racism in health care services and a lack of culturally appropriate and coordinated cancer care [27,28,29]. Corresponding disparities in cancer health outcomes for Indigenous Australians have persisted over decades, prompting a strong focus of achieving equity in cancer outcomes for Indigenous Australians within the new Australian Cancer Plan and the Aboriginal and Torres Strait Islander Cancer Plan [30,31].

Previous studies have included data on NT Indigenous Australian childhood cancer outcomes utilizing sources such as the national Australian Childhood Cancer Registry and hospital records reviews [32,33,34]. However, no study to date has focused on cancer subtypes and outcomes among NT Indigenous Australian children as a separate sub-cohort. The current study identified a temporal reduction in the incidence of childhood cancer in non-Indigenous children, whilst cancer incidence remained unchanged in Indigenous Australian children. Consistent with the previous literature, overall survival of Indigenous Australian children in the NT was lower compared with the non-Indigenous cohort [32,33,34]. However, overall survival for Indigenous Australian children in the NT improved from 1990 to 2017, indicating that Indigenous Australian children in the NT may be beginning to benefit from advancements in clinical cancer care made over the past three decades.

A major strength of the current study is the collection of high-quality data from the SACR and NTCR over 28 years, with strict inclusion criteria, representing a rigorous process to gain invaluable insight into childhood cancer trends in SA and the NT. However, further study is needed to collect detailed socio-economic data and data on perinatal lifestyle choices to understand the underlying factors that drive early carcinogenesis. The registries lack details of treatment and stage of disease at diagnosis which could impact the interpretation of survival data. Moreover, limited sample size of the Indigenous cohort with a lack of these data from SA may have influenced statistical power, rending limitations to in-depth analyses, disease subtype-specific analyses and interpretations of data. Improved Indigenous identification within health data across all Australian jurisdictions is needed to ensure visibility of Indigenous Australians within cancer registries. Increased engagement between cancer registries, Indigenous Australian communities and Indigenous health experts are needed to ensure these data sources can be best utilized to influence clinical practice and policy to close the gap for Indigenous Australians affected by cancer.

## 5. Conclusions

Overall, this study demonstrates that the incidence of childhood cancers has remained largely unchanged across 28 years in SA and the NT. Improved survival rates over time were observed in SA and amongst Indigenous Australian children of the NT. Nevertheless, cancer survival rates for Indigenous Australian children remain lower than non-Indigenous Australian children, indicating a continuing need for new interventions to ensure equity in cancer outcomes for this population. Moreover, these outcomes provide further impetus towards reducing the risk of childhood cancer by supporting mothers and young women of reproductive age who intend to become mothers to improve health literacy and lifestyle factors. There is a clear need for a concerted global effort aimed at understanding the risk factors that serve as drivers of early carcinogenesis; this information would support the development of biomarkers to aid in early detection of cancers, at a stage when a cure is possible.

## Figures and Tables

**Table 1:** Children’s characteristics, Indigenous status and era for SA and NT.

	SA & NT		SA		NT	
	*N* = 895		*N* = 753		*N* = 142	
	N	%	N	%	N	%
Age, years, median (IQR)	5	(2–13)	4	(2–12)	7	(2–16)
Age, years						
<5	433	48.4	377	50.1	56	39.4
≥5	462	51.6	376	49.9	86	60.6
Sex						
Female	398	44.5	342	45.4	56	39.4
Male	497	55.5	411	54.6	86	60.6
Indigenous status						
Non-Indigenous	100	11.2	-	-	100	70.4
Indigenous	39	4.4	-	-	39	27.5
Missing	756	84.5	753	100	3	2.1
Era						
1990–1999	300	33.5	250	33.2	50	35.2
2000–2009	312	34.9	270	35.9	42	29.6
2010–2017	283	31.6	233	30.9	50	35.2
Cancer subtype						
Lymphocytic leukemias	555	62.0	483	64.1	72	50.7
Nephroblastoma	84	9.4	70	9.3	14	9.9
Neuroblastoma	62	6.9	55	7.3	7	4.9
Ewing Sarcoma	48	5.4	40	5.3	8	5.6
Osteosarcoma	39	4.4	33	4.4	6	4.2
Retinoblastoma	37	4.1	33	4.4	4	2.8
Hepatoblastoma	23	2.6	18	2.4	5	3.5
Rhabdomyosarcoma	38	4.2	14	1.9	24	16.9
Medulloblastoma	9	1.0	7	0.9	2	1.4

Note: Number and percentages are reported unless stated otherwise; Indigenous data are only available for NT; IQR Interquartile range.

**Table 2:** Incidence rates, incidence rate ratios and 95% CI (Poisson regression model) for age, sex, era and cancer subtypes between SA and NT.

		SA & NT		SA			NT		
		N = 895		N = 753			N = 142		
	*IR (95% CI)	IRR (95% CI)	*p*-Value	*IR (95% CI)	IRR (95% CI)	*p*-Value	*IR (95% CI)	IRR (95% CI)	*p*-Value
Overall	193.04 (180.60–206.12)			189.37 (176.09–203.39)			215.16 (181.23–253.60)		
Age (years)									
<5	379.02 (344.15–416.45)	Reference	-	391.99 (353.41–433.62)	Reference		309.97 (234.15–402.52)	Reference	
≥5	132.23 (120.45–144.86)	0.35 (0.31–0.40)	<0.001	124.73 (112.44–138.00)	0.32 (0.28–0.37)	<0.001	179.42 (143.52–221.59)	0.58 (0.41–0.81)	<0.01
Sex									
Female	176.31 (159.41–194.51)	Reference	-	176.38 (158.18–196.10)	Reference	-	175.91 (132.88–228.43)	Reference	-
Male	208.92 (190.95–228.12)	1.19 (1.04–1.35)	0.01	201.74 (182.70–222.22)	1.14 (0.99–1.32)	0.07	251.74 (201.36–310.90)	1.43 (1.02–2.01)	0.04
Era									
1990–1999	181.67 (161.70–203.44)	Reference	-	175.12 (154.08–198.23)	Reference	-	223.50 (165.89–294.66)	Reference	-
2000–2009	190.55 (169.99–212.91)	1.06 (0.91–1.25)	0.44	193.17 (170.82–217.64)	1.12 (0.94–1.33)	0.19	175.27 (126.32–236.91)	0.78 (0.52–1.18)	0.25
2010–2017	210.00 (186.24–235.94)	1.15 (0.98–1.35)	0.09	202.43 (177.27–230.16)	1.15 (0.96–1.37)	0.13	254.28 (188.73–335.24)	1.14 (0.77–1.68)	0.52
Cancer subtype									
Lymphocytic leukemias	119.71 (109.95–130.09)			121.47 (110.88–132.80)	-	-	109.10 (85.36–137.39)	-	0.43
Nephroblastoma	18.12 (14.45–22.43)			17.60 (13.72–22.24)	-	-	21.21 (11.60-35.59)	-	0.63
Neuroblastoma	13.37 (10.25–17.14)			13.83 (10.42–18.00)			10.61 (4.26–21.85)		0.63
Ewing Sarcoma	10.35 (7.63–13.73)			10.06 (7.19–13.70)	-	-	12.12 (5.23–23.88)	-	0.78
Osteosarcoma	8.41 (5.98–11.50)			8.30 (5.71–11.67)	-	-	9.09 (3.34–19.79)	-	>0.9
Retinoblastoma	7.98 (5.62–11.00)			8.30 (5.71–11.67)			6.06 (1.65–15.52)		0.72
Hepatoblastoma	4.96 (3.14–7.44)			4.53 (2.68–7.15)			7.58 (2.46–17.68)		0.46
Rhabdomyosarcoma	8.20 (5.80–11.25			3.52 (1.92–5.91)			36.37 (2.33–54.11)		<0.001
Medulloblastoma	1.94 (0.89–3.69			1.76 (0.71–3.63)			3.03 (0–10.95)		0.83

*IR (incidence rate) is incidence per 100,000 residents. IRRs (incidence rate ratios) were not reported for cancer subtypes. *p* Values for cancer subtype are based on Chi-square test and differentiated incidenece rates between SA and NT. CI confidence interval.

**Table 3:** Incidence rates, incidence rate ratios and 95% CI (Poisson regression model) for age, sex, era and cancer subtypes between Indigenous and non-Indigenous children in NT.

	Non-Indigenous			Indigenous		
	N = 100			N = 39		
	*IR (95% CI)	IRR (95% CI)	*p*-Value	*IR (95% CI)	IRR (95% CI)	*p*-Value
Overall	226.28 (184.11–275.22)			158.94 (113.02–217.28)		
Age (years)	-			-		
<5	299.13 (210.62–412.32)	Reference	-	277.64 (161.74–444.53)	Reference	-
≥5	197.96 (152.12–253.28)	0.66 (0.44–0.99)	0.046	119.47 (74.87–180.89)	0.43 (0.22–0.81)	<0.01
Sex						
Female	181.15 (128.19–248.64)	Reference	-	156.19 (153.30–159.11)	Reference	-
Male	267.06 (204.75–342.36)	1.47 (0.98–2.21)	0.06	242.33 (238.51–246.21)	1.09 (0.58–2.06)	0.77
Era						
1990–1999	278.32 (199.73–377.58)	Reference	-	110.04 (50.32–208.89)	Reference	-
2000–2009	162.92 (104.38–242.41)	0.58 (0.35–0.97)	0.04	195.62 (111.82–317.68)	1.78 (0.79–4.02)	0.17
2010–2017	237.59 (165.49–330.44)	0.85 (0.54–1.34)	0.49	171.17 (93.58–287.19)	1.56 (0.67–3.59)	0.30
Cancer subtype						
Lymphocytic leukemias	101.83 (74.27–136.25)	-	-	105.96 (69.22–155.26)	-	-
Nephroblastoma	24.89 (12.43–44.54)	-	-	12.23 (2.52–35.73)	-	-
Neuroblastoma	9.05 (2.47–23.17)	-	-	12.23 (2.52–35.73)	-	-
Ewing Sarcoma	13.58 (4.98–29.55)	-	-	4.08 (0–22.71)	-	-
Osteosarcoma	6.79 (1.40–19.84)	-	-	12.23 (2.52–35.73)	-	-
Retinoblastoma	9.05 (2.47–23.17)	-	-	-	-	-
Hepatoblastoma	6.79 (1.40–19.84)	-	-	8.15 (0–29.44)	-	-
Rhabdomyosarcoma	52.04 (32.99–78.09)	-	-		-	-
Medulloblastoma	2.26 (0–12.61)	-	-	4.08 (0–22.71)	-	-

*IR (incidence rate) is incidence per 100,000 residents; 2011 census population was used for incidence rates. IRRs (incidence rate ratios) were not reported for cancer subtypes. CI confidence interval.

**Table 4:** 5-year survivals for primary subtypes of cancer between SA and NT.

Cancer Subtypes	SA & NT	SA	NT
	N = 895	N = 753	N = 142
		Proportion (95% CI)	Proportion (95% CI)
Overall	75% (72–78)	77% (74–80)	62% (51–71)
Lymphocytic leukemias	78% (74–82)	82% (78–85)	62% (49–72)
Nephroblastoma	86% (75–93)	89% (79–95)	86% (54–96)
Neuroblastoma	53% (38–66)	59% (43–71)	54% (13–83)
Ewing Sarcoma	54% (38–67)	55% (38–69)	50% (15–77)
Osteosarcoma	54% (37–69)	62% (42–76)	33% (5–68)
Retinoblastoma	100	100	100
Hepatoblastoma	82% (53–94)	89% (61–97)	80% (20–97)
Rhabdomyosarcoma	77% (55–89)	69% (37–87)	91% (70–98)
Medulloblastoma	60% (20–85)	63% (14–89)	50% (1–91)

**Table 5:** Hazard ratios and 95% CI (Cox Proportional hazards model) for age, sex and era for SA and NT.

	All		SA		NT	
	N = 895		N = 753		N = 142	
	HR (95% CI)	*p* Value	HR (95% CI)	*p* Value	HR (95% CI)	*p* Value
Age (years)	1.07 (1.05–1.09)	<0.001	1.08 (1.05–1.10)	<0.001	1.03 (0.98–1.08)	0.22
Age (years)						
<5	Reference	-	Reference	-	Reference	
≥5	1.87 (1.40–2.51)	<0.001	2.00 (1.47–2.72)	<0.001	1.57 (0.84–2.94)	0.16
Sex						
Female	Reference	-	Reference	-	Reference	-
Male	1.03 (0.78–1.36)	0.82	0.98 (0.73–1.32)	0.90	0.93 (0.52–1.66)	0.79
Indigenous status						
Non-Indigenous	-	-	-	-	Reference	
Indigenous	-	-	-	-	3.42 (1.92–6.10)	<0.001
Era						
1990–1999	Reference	-	Reference	-	Reference	-
2000–2009	0.36 (0.27–0.50)	<0.001	0.53 (0.38–0.73)	<0.001	0.78 (0.40–1.51)	0.46
2010–2017	0.31 (0.31–0.44)	<0.001	0.44 (0.28–0.68)	<0.001	0.50 (0.24–1.05)	0.07

Note: Data on Indigenous peoples are only available for NT. HR hazard ratio; CI confidence interval.

**Table 6:** 5-year survivals for primary subtypes of cancer between Indigenous and Non-Indigenous peoples in NT.

Cancer Subtypes	Non-Indigenous	Indigenous
	N = 100	N = 39
	Proportion (95% CI)	Proportion (95% CI)
Overall	79% (69–85)	38% (22–53)
Lymphocytic leukemias	70% (54–82)	45% (25–63)
Nephroblastoma	91% (51–99)	67% (5–95)
Neuroblastoma	100	-
Ewing Sarcoma	50% (11–80)	-
Osteosarcoma	33% (1–77)	33% (1–77)
Retinoblastoma	100	-
Hepatoblastoma	100	50% (1–91)
Rhabdomyosarcoma	91% (69–97)	-
Medulloblastoma	100	-

**Table 7:** Hazard ratios and 95% CI (Cox Proportional hazards model) for age, sex and era between Indigenous and non-Indigenous children in NT.

	Non-Indigenous		Indigenous	
	N = 100		N = 39	
	HR (95% CI)	*p* Value	HR (95% CI)	*p* Value
Age (years)	1.02 (0.97–1.09)	0.42	1.06 (0.99–1.13)	0.08
Age (years)				
<5	Reference	-	Reference	-
≥5	1.38 (0.57–3.32)	0.48	2.34 (0.96–5.70)	0.06
Sex				
Female	Reference	-	Reference	-
Male	1.17 (0.50–2.74)	0.72	1.06 (0.47–2.41)	0.89
Era				
1990–1999	Reference	-	Reference	-
2000–2009	0.74 (0.0.28–1.93)	0.54	0.35 (0.13–0.93)	0.04
2010–2017	0.47 (0.0.17–1.33)	0.17	0.29 (0.10–0.83)	0.02

HR hazard ratio; CI confidence interval.

## Data Availability

Authors are unable to provide this data owing to the Ethics approval being granted on the premise that the (South Australian and the Northern Territory Cancer Registry) data will not be released to a third party.

## Supplementary Materials

The following supporting information can be downloaded at: https://www.mdpi.com/article/10.3390/cancers16112057/s1, Figure S1: Trend in incidence rates by sex and era between two age groups (<5 and ≥5) across cancer subtypes in SA and NT between 1990–2017; Figure S2: Incidence rate ratios (IRR) and 95% Confidence Intervals (CI) (Poisson regression model) for sex, era and age groups by primary subtype in SA and NT. Figure S3: Hazard ratios (HR) and 95% Confidence Intervals (CI) (Cox proportional hazards model) for sex, era and age groups by primary subtype in SA and NT. Table S1: Hazard ratios (HR) and 95% Confidence Intervals (CI) (Cox Proportional hazards model) for age, sex and era for SA and NT in Lymphocytic leukemias. Table S2: Hazard ratios (HR) and 95% Confidence Intervals (CI) (Cox Proportional hazards model) for age, sex and era for SA and NT in Nephroblastoma. Table S3: Hazard ratios (HR) and 95% Confidence Intervals (CI) (Cox Proportional hazards model) for age, sex and era for SA and NT in Rhabdomyosarcoma.
