# Supplementary material for: Childhood Cancer Incidence and Survival in South Australia and the Northern Territory, 1990–2017, with Emphasis on Indigenous Peoples
Source: Cancers (Basel). 2024 May 29;16(11):2057. doi: 10.3390/cancers16112057 (PMC11171054; doi:10.3390/cancers16112057)
Supplement: Supplementary file 1 [file cancers-16-02057-s001.zip › cancers-3024659-supplementary.pdf]

Supplementary Figure S1. Trend in incidence rates by sex and era between two age groups (<5 and ≥5) across cancer subtypes in SA and NT between 1990-2017

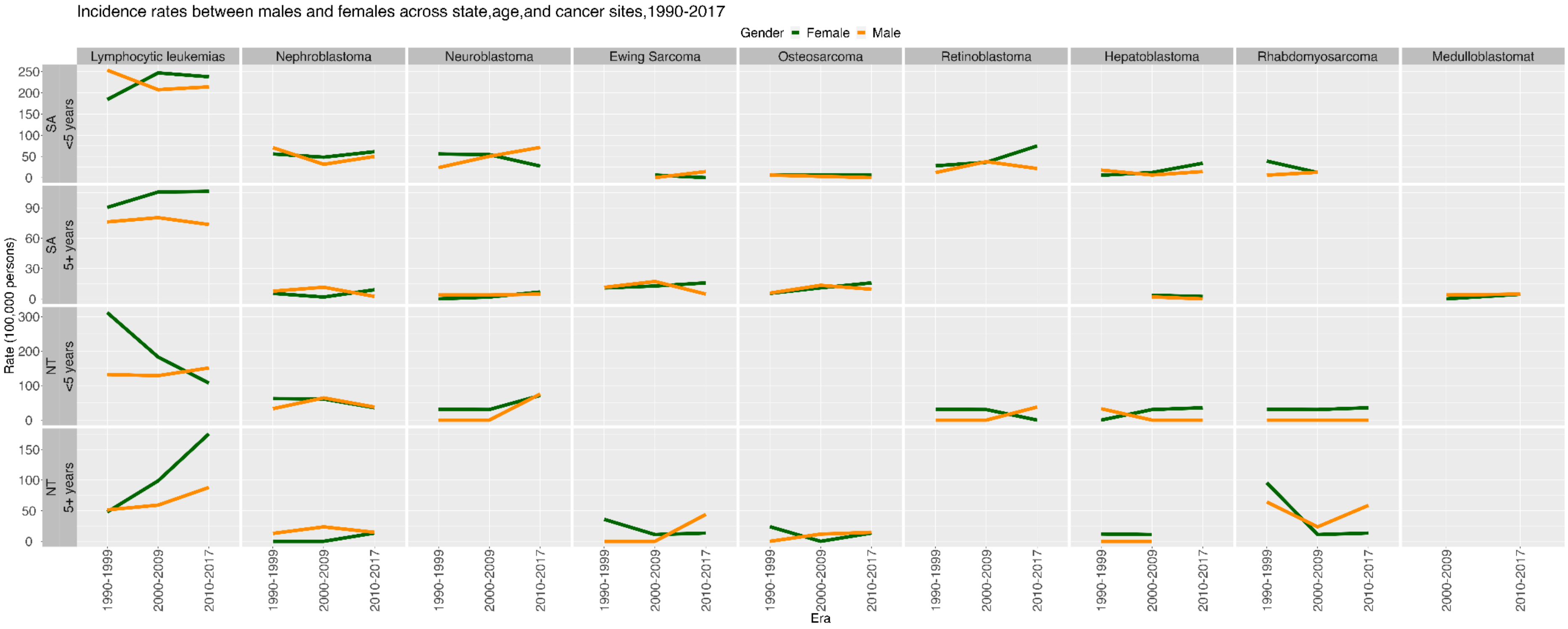

Supplementary Figure S2. Incidence rate ratios (IRR) and 95% Confidence Intervals (CI) (Poisson regression model) for sex, era and age groups by primary subtype in SA and NT

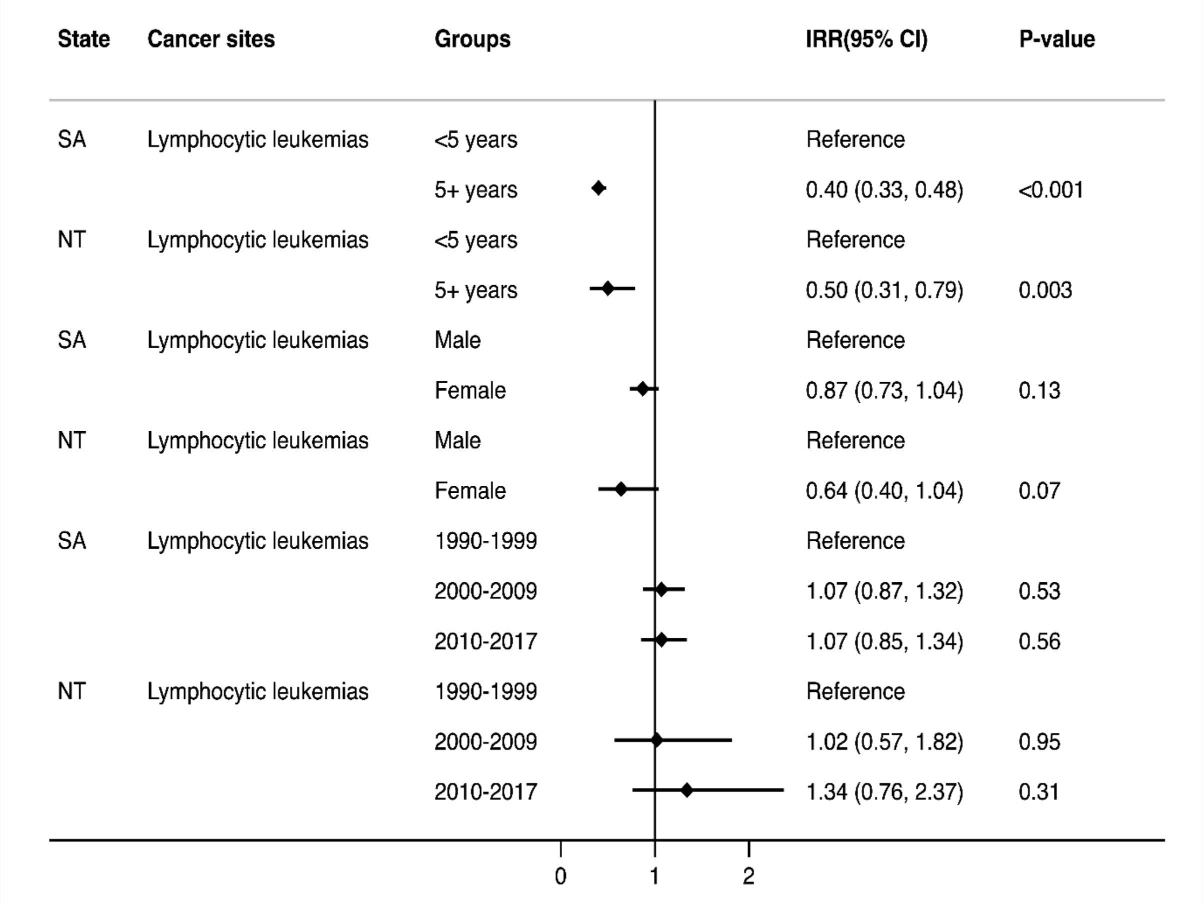

Supplementary Figure S3. Hazard ratios (HR) and 95% Confidence Intervals (CI) (Cox proportional hazards model) for sex, era and age groups by primary subtype in SA and NT

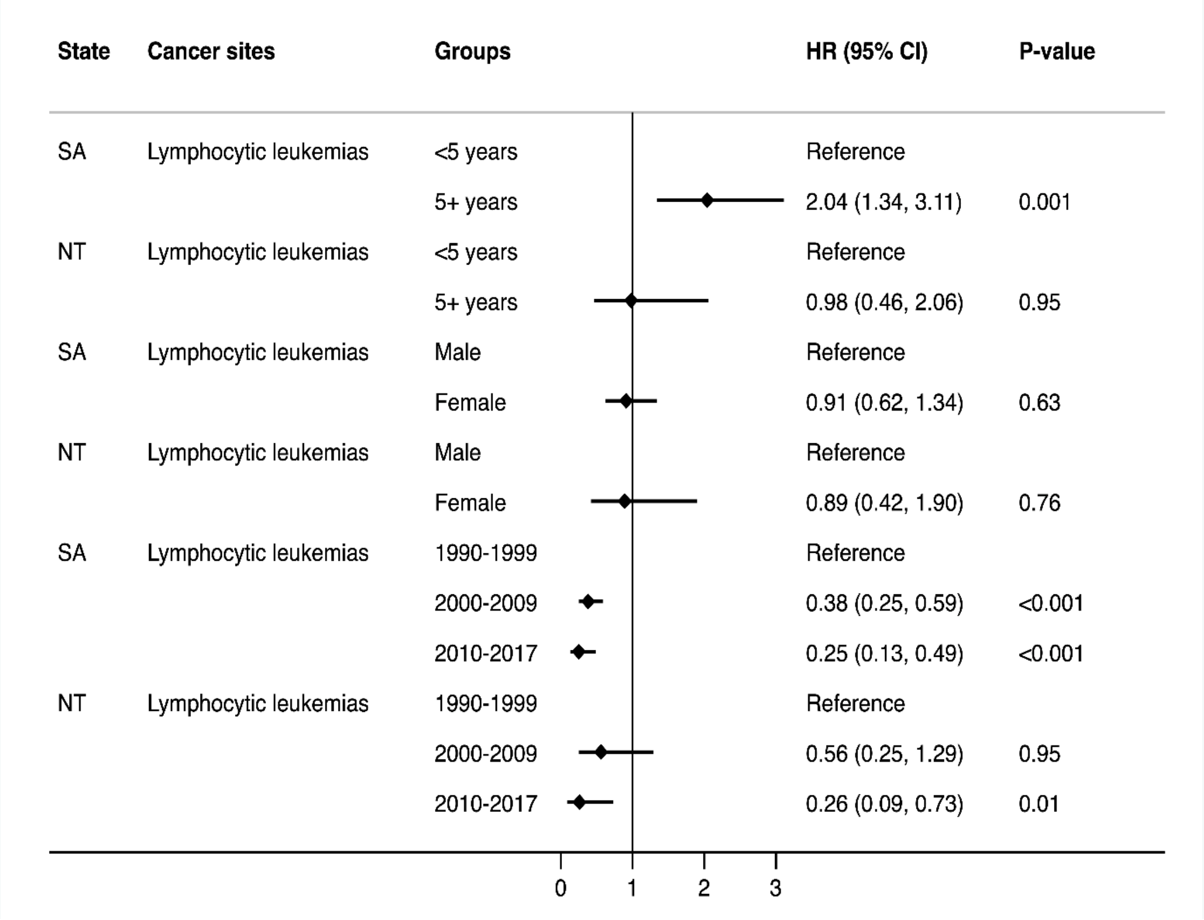

Supplementary Table S1. Hazard ratios (HR) and 95% Confidence Intervals (CI) (Cox Proportional hazards model) for age, sex and era for SA and NT in Lymphocytic leukemias

	All		SA		NT	
	N=555		N=483		N=72	
	HR (95% CI)	P Value	HR (95% CI)	P Value	HR (95% CI)	P Value
**Age (years)**	1.07 (1.03-1.10)	<0.001	1.08 (1.05-1.12)	<0.001	1.01 (0.95-1.08)	0.68
**Age (years)**						
<5	Reference	-	Reference	-	Reference	
≥5	1.67 (1.14-2.44)	<0.01	2.04 (1.34-3.11)	<0.01	0.98 (0.46-2.06)	0.95
**Sex**						
Female	Reference	-	Reference	-	Reference	-
Male	0.90 (0.63-1.29)	0.57	0.91 (0.62-1.34)	0.63	0.89 (0.42-1.90)	0.76
**Indigenous status**						
Non-Indigenous	-	-	-	-	Reference	
Indigenous	-	-	-	-	2.09 (0.99-4.41)	0.05
**Era**						
1990-1999	Reference	-	Reference	-	Reference	-
2000-2009	0.36 (0.24-0.53)	<0.001	0.38 (0.25-0.59)	<0.001	0.56 (0.25-1.29)	0.18
2010-2017	0.24 (0.14-0.42)	<0.001	0.25 (0.13-0.49)	<0.001	0.26 (0.09-0.73)	0.01

Note: Data on Indigenous peoples are only available for NT

Supplementary Table S2. Hazard ratios (HR) and 95% Confidence Intervals (CI) (Cox Proportional hazards model) for age, sex and era for SA and NT in Nephroblastoma

	**All**		**SA**		**NT**	
	**N=84**		**N=70**		**N=14**	
	**HR (95% CI)**	**P Value**	**HR (95% CI)**	**P Value**	**HR (95% CI)**	**P Value**
**Age (years)**	1.22 (1.05-1.41)	<0.01	1.16 (0.97-1.39)	0.11	1.75 (1.01-3.05)	0.048
**Age (years)***						
<5	Reference	-	Reference	-	Reference	
≥5	2.06 (0.55-7.66)	0.28	0.98 (0.19-5.07)	0.98	-	-
**Sex**						
Female	Reference	-	Reference	-	Reference	-
Male	0.82 (0.22-3.04)	0.76	1.35 (0.30-6.05)	0.69	-	-
**Indigenous status**						
Non-Indigenous	-	-	-	-	Reference	
Indigenous	-	-	-	-	4.49 (0.28-72.83)	0.29
**Era**						
1990-1999	Reference	-	Reference	-	Reference	-
2000-2009	1.29 (0.32-5.17)	0.72	0.76 (0.14-4.15)	0.75	-	-
2010-2017	0.38 (0.04-3.40)	0.39	0.44 (0.05-3.96)	0.46	-	-

*Note: Data on Indigenous peoples are only available for NT. * NT estimates were not provided due to small number of cases*

Supplementary Table S3. Hazard ratios (HR) and 95% Confidence Intervals (CI) (Cox Proportional hazards model) for age, sex and era for SA and NT in Rhabdomyosarcoma

	All		SA		NT	
	N=38		N=14		N=24	
	HR (95% CI)	P Value	HR (95% CI)	P Value	HR (95% CI)	P Value
**Age (years)**	0.94 (0.86-1.04)	0.21	1.47 (0.98-2.21)	0.07	1.11 (0.69-1.78)	0.66
**Age (years)***						
<5	Reference	-	Reference	-	Reference	
≥5	0.44 (0.10-1.95)	0.28	2.30 (0.23-22.52)	0.48	-	-
**Sex**						
Female	Reference	-	Reference	-	Reference	-
Male	0.46 (0.10-2.05)	0.31	0.28 (0.05-1.65)	0.16	0.76 (0.05-12.28)	0.85
**Indigenous status**						
Non-Indigenous	-	-	-	-	Reference	
Indigenous	-	-	-	-	-	-
**Era***						
1990-1999	Reference	-	Reference	-	Reference	-
2000-2009	-	-	-	-	-	-
2010-2017	-	-	-	-	-	-

*Note: Data on Indigenous peoples are only available for NT. * Estimates were not provided due to small number of cases*
